# Doxycycline Reduces Plasma VEGF-C/sVEGFR-3 and Improves Pathology in Lymphatic Filariasis

**DOI:** 10.1371/journal.ppat.0020092

**Published:** 2006-09-15

**Authors:** Alexander Yaw Debrah, Sabine Mand, Sabine Specht, Yeboah Marfo-Debrekyei, Linda Batsa, Kenneth Pfarr, John Larbi, Bernard Lawson, Mark Taylor, Ohene Adjei, Achim Hoerauf

**Affiliations:** 1 Institute for Medical Microbiology, Immunology, and Parasitology, University of Bonn, Bonn, Germany; 2 Kumasi Centre for Collaborative Research in Tropical Medicine, Kumasi, Ghana; 3 Department of Theoretical and Applied Biology, Kwame Nkrumah University of Science and Technology, Kumasi, Ghana; 4 Liverpool School of Tropical Medicine, Liverpool, United Kingdom; 5 School of Medical Sciences, Kwame Nkrumah University of Science and Technology, Kumasi, Ghana; Northwestern University Medical School, United States of America

## Abstract

Lymphatic filariasis is a disease of considerable socioeconomic burden in the tropics. Presently used antifilarial drugs are able to strongly reduce transmission and will thus ultimately lower the burden of morbidity associated with the infection, however, a chemotherapeutic principle that directly induces a halt or improvement in the progression of the morbidity in already infected individuals would constitute a major lead. In search of such a more-effective drug to complement the existing ones, in an area endemic for bancroftian filariasis in Ghana, 33 microfilaremic and 18 lymphedema patients took part in a double-blind, placebo-controlled trial of a 6-wk regimen of 200 mg/day doxycycline. Four months after doxycycline treatment, all patients received 150–200 μg/kg ivermectin and 400 mg albendazole. Patients were monitored for *Wolbachia* and microfilaria loads, antigenemia, filarial dance sign (FDS), dilation of supratesticular lymphatic vessels, and plasma levels of lymphangiogenic factors (vascular endothelial growth factor-C [VEGF-C] and soluble vascular endothelial growth factor receptor-3 [(s)VEGFR-3]). Lymphedema patients were additionally monitored for stage (grade) of lymphedema and the circumferences of affected legs. *Wolbachia* load, microfilaremia, antigenemia, and frequency of FDS were significantly reduced in microfilaremic patients up to 24 mo in the doxycycline group compared to the placebo group. The mean dilation of supratesticular lymphatic vessels in doxycycline-treated patients was reduced significantly at 24 mo, whereas there was no improvement in the placebo group. Preceding clinical improvement, at 12 mo, the mean plasma levels of VEGF-C and sVEGFR-3 decreased significantly in the doxycycline-treated patients to a level close to that of endemic normal values, whereas there was no significant reduction in the placebo patients. The extent of disease in lymphedema patients significantly improved following doxycycline, with the mean stage of lymphedema in the doxycycline-treated patients being significantly lower compared to placebo patients 12 mo after treatment. The reduction in the stages manifested as better skin texture, a reduction of deep folds, and fewer deep skin folds. In conclusion, a 6-wk regimen of antifilarial treatment with doxycycline against W. bancrofti showed a strong macrofilaricidal activity and reduction in plasma levels of VEGF-C/sVEGFR-3, the latter being associated with amelioration of supratesticular dilated lymphatic vessels and with an improvement of pathology in lymphatic filariasis patients.

## Synopsis

Lymphatic filariasis, caused by filarial worms, is transmitted by mosquitoes. The infection leads to pathology such as edema of the legs (lymphedema) or the scrotum (hydrocele). About 120 million people are estimated to be infected, and 1.2 billion are at risk of infection. The currently used drugs (diethylcarbamazine [DEC] or ivermectin plus albendazole) to treat the disease are able to kill most of the larval stage (microfilariae) in the blood but have either no (ivermectin) or partial (DEC) effect on the adult worms that cause the pathology. They also do not sufficiently halt the progression of pathology, such as, lymph vessel dilation, hydrocele, and lymphedema.

In search of a more effective drug capable of killing the adult worms, and also of halting the progression of the disease in already infected individuals, the authors recruited, in an endemic area in Ghana, 33 people who were infected with the worm and microfilaremic but had not yet developed the disease, as well as 18 lymphedema patients, and treated them with either 200 mg/d doxycycline or matching placebo for 6 wk. The findings presented here reveal that doxycycline is able to kill the adult worms, improves lymphatic vessel dilation, and ameliorates the conditions of lymphedema patients significantly. This suggests that doxycycline can be used to treat lymphatic filarial infections and pathology, making doxycycline the first drug already approved for human use and considered for use as an adjunct to current control programs, which in addition to parasite control achieves improvement of the quality of life of persons with pathology.

## Introduction

Bancroftian filariasis is a mosquito-transmitted parasitic disease of humans characterized by lymphangitis, hydrocele, lymphedema, and elephantiasis, and is one of the most common causes of global disability [1]. The disease has been considered to be potentially eradicable due to the fact that antifilarial drugs currently used could break the cycle of transmission in endemic areas. The goals of the current global lymphatic filariasis (LF) elimination program are (1) to reduce microfilaremia levels with filaricidal drugs to a level that is too low to sustain transmission of filarial parasites to humans; and (2) to reduce the morbidity associated with chronic filarial disease [2].

The antifilarial drugs currently used, diethylcarbamazine (DEC) and ivermectin, are predominantly active against microfilariae (MF), with DEC showing partial activity against adult worms [3]. Mass drug applications using DEC, which acts on the adult worms that are believed to be major inducers of the lymphatic pathology, have been reported to lead to an improvement of pre-existing lymphedema and hydrocele [4,5]; similar findings were observed in a controlled treatment study using different regimens of DEC [6]. However, these results are not unequivocal, since several other studies could not confirm these findings even when DEC was administered for 12 d instead of the usual single dose of the mass drug administration (MDA) [7–12]. This inconsistency was also acknowledged in a conference by experts of the “Filariasis Community of Scientists in Association with an LF Research Forum” convened in Philadelphia, December 2003 [13].

Thus, a drug with stronger macrofilaricidal activity than reported for DEC, which simultaneously has ameliorating effects on lymphatic vessels, could considerably improve the prospect of developing a treatment for lymphatic pathology. One aspect of the biology of filarial nematodes that could be exploited in the effort to advance the elimination program is the presence of the endosymbiotic *Wolbachia* found in most filarial species, including W. bancrofti [14]. Recent studies of symbiotic *Wolbachia* organisms suggest that these bacteria are important both as chemotherapeutic targets and disease-causing organisms [14]. In a laboratory model of onchocercal keratitis, *Wolbachia* was shown to mediate neutrophil infiltration and stromal haze when a worm extract including *Wolbachia* antigens was injected into the eyes of mice [15–17].

The events that lead to the development of chronic pathology in LF are not fully understood, but the immune responses of the human host to the parasites are believed to play a significant role in determining the pathological manifestations of disease in infected individuals [18–21].

The lymphatic vascular system is important for immune surveillance, tissue fluid homeostasis, and fat absorption [22,23]. Perturbations in the maintenance and function of the lymphatic system can lead to a variety of pathological disorders, including lymphatic dilation and lymphedema [24–26].

Recent studies on the molecular mechanisms controlling the lymphatic vessels have shown that vascular endothelial growth factors C (VEGF-C) and VEGF-D specifically control lymphangiogenesis in humans [27,28] by activating the VEGF receptor-3 (VEGFR-3) [29–32], which is principally restricted to the lymphatic endothelium in adults [33,34]. In animal models, overexpression of VEGF-C in the skin of transgenic mice resulted in lymphatic endothelial proliferation and dilation of lymph vessels [32] with a resemblance to lymphatics infected with filarial parasites [35]. Additional evidence for the role of VEGF-C/VEGF-D/VEGFR-3 in the pathogenesis of lymphatic dilation and lymphedema stems from experimental studies in transgenic mice with skin specific overexpression of soluble VEGFR-3 (sVEGFR-3) using a *keratin 14* transgenic promoter [36]. In this genetic model, sVEGFR-3 is secreted at high levels by basal epidermal keratinocytes and binds the lymphangiogenesis factors VEGF-C and VEGF-D, thereby preventing them from activating membrane-bound VEGFR-3 on lymphatic endothelium [36]. These K14/ sVEGFR-3 transgenic mice lack a functional cutaneous lymphatic system and are characterized by lymphedema formation in the skin. The expression of VEGF-C has been shown to be up-regulated by proinflammatory cytokines like interleukin (IL)-1B and tumor necrosis factor (TNF), suggesting that proinflammatory cytokines could affect the lymphatic vessels via VEGF-C [37].

Studies in animal models have shown that *Wolbachia*–derived molecules from Brugia spp. induced proinflammatory cytokines, including TNF and IL-1B [38]. Soluble extracts of *Brugia* and Onchocerca volvulus adult and microfilarial worms were also found to stimulate human peripheral mononuclear cells in vitro, resulting in the production of TNF, IL-1, granulocyte-macrophage colony-stimulating factor (GM-CSF), and IL-10 [39,40]. This stimulation was not achieved using extracts from *Acanthocheilonema viteae,* a filarial species naturally devoid of *Wolbachia,* and, importantly, with O. volvulus extracts from patients that had been treated with doxycycline to deplete *Wolbachia* from the worms [41]. Thus it was concluded that in those filarial species that contain these endosymbionts, *Wolbachia* are the major stimulating principle for proinflammatory cytokines such as TNF. From this it can be further hypothesized that exposure of host cells to *Wolbachia* from worms (either from dying adult worms or incoming L3/4 larvae, or from the proportion of degenerating embryos that are constantly released) may induce the production of lymphangiogenic factors such as VEGF-C by endothelial cells in LF patients.

Treatment with doxycycline for 8 wk is well tolerated in humans and has shown macrofilaricidal activity [42]. Doxycycline is also effective in depleting *Wolbachia* from both MF and adult filarial worms of W. bancrofti and O. volvulus [43,44]. Of importance for the present study, *Wolbachia* depletion by doxycycline was associated with a reduction of pretreatment elevated levels of proinflammatory cytokines such as TNF from plasma [45]. Together, these data allow the hypothesis that targeting *Wolbachia* by doxycycline may ameliorate filarial pathology through down-regulation of proinflammatory cytokines and VEGF-C/VEGFR-3. However, no studies to date have analyzed *Wolbachia-*induced secretion of VEGF-C and its receptor on lymphatic vessel dilation among human populations with lymphatic filarial disease.

The aims of this study were to assess (1) a 6-wk course of doxycycline for macrofilaricidal activity; (2) the effect of targeting the endosymbiotic *Wolbachia* in filarial worms on the levels of VEGF-C and its soluble receptor VEGFR-3 and on dilation of the supratesticular lymphatic vessels in microfilaremic patients; and (3) the effect of doxycycline treatment on clinical manifestations associated with filarial lymphedema. To address these aims, we recruited microfilaremic and lymphedema patients in an endemic area in Ghana and treated them with 200 mg/d doxycycline for 6 wk. Lymphedema patients were followed up at 4 and 12 mo, whereas microfilaremic patients were re-examined at 4, 12, and 24 mo after the start of doxycycline treatment. We report that 6 wk of doxycycline had a strong macrofilaricidal activity; and targeting the endosymbiotic *Wolbachia* in filarial worms resulted in the reduction of plasma levels of VEGF-C/sVEGFR-3, which were associated with amelioration of dilated supratesticular lymphatic vessels and improvement of lymphedema in lymphatic filariasis patients.

## Results

We treated a total 95 patients with bancroftian filariasis (76 microfilaremic and 19 lymphedema) with doxycycline or matching placebo for 6 wk. The treatment was well tolerated, with eight doxycycline and ten placebo patients experiencing adverse reactions, which in no case required a treatment stop. The adverse reactions arose on days 2 and 3 after commencement of treatment in both groups. The adverse reactions experienced by the eight doxycycline-treated patients included headache, dizziness, diarrhea, and itching skin. No adverse event went beyond 3 d or required more intervention than application of paracetamol tablets, oral dehydration salt, and ointment. The adverse reactions experienced by the ten placebo patients included headache, diarrhea, and painful scrotum. Again no event lasted for more than 3 d, except for one microfilaremic patient who had a painful scrotum for 6 d. In this group, the side effects were also treated with paracetamol tablets and oral dehydration salt. There was no significant difference between the doxycycline and placebo groups.

**Figure 1:**
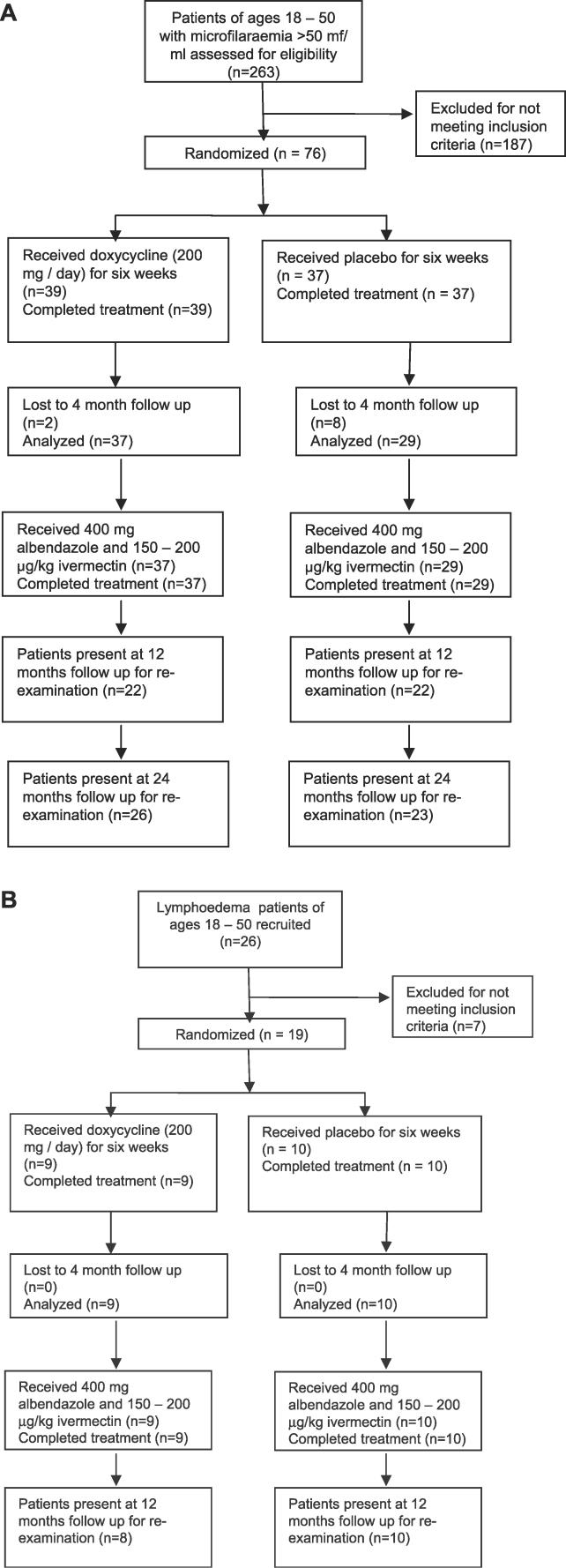
Flowchart of Patient Participation (A) Trial profile of microfilaremic patients. Of the 76 patients, 33 (17 doxycycline and 16 placebo) patients were present at all time points. (B) Trial profile of lymphedema patients. Of the 19 patients, 18 (eight doxycycline and ten placebo) patients were present at all time points.

Thirty-three (17 doxycycline and 16 placebo) microfilaremic patients and eighteen lymphedema patients (8 doxycycline and 10 placebo) were present at all time points (Table 1), and the analyses are based on these patients. In all the analyses, only participants present at all time points were included. Nonetheless, the significant differences observed were not affected when all patients (including the dropouts at the follow-up time points as shown in Figure 1A and 1B) were included in the analyses (unpublished data).

**Table 1:**
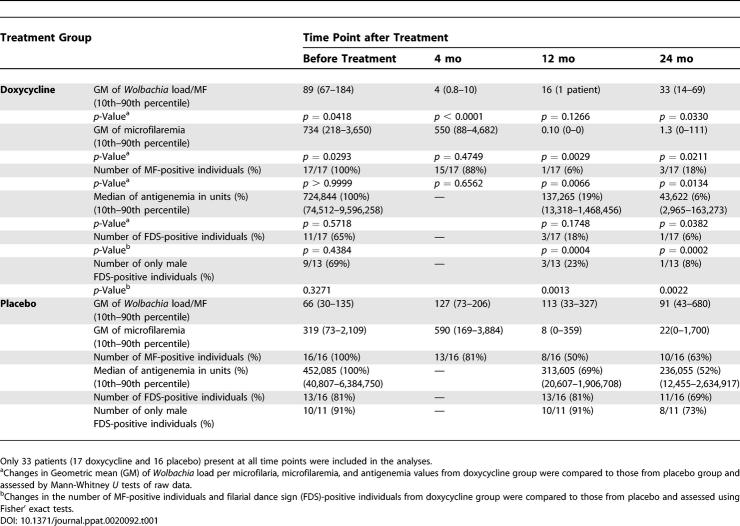
Primary Variables Measured before and after Treatment in Microfilaremic Patients

### Primary Outcome Analysis in Microfilaremic Patients

Table 1 shows the changes in *Wolbachia* level, microfilaremia, and antigenemia from baseline and at follow-up time points. In all the analyses, only participants present at all time points (17 doxycycline and 16 placebo) were included. Following doxycycline treatment, *Wolbachia* levels in MF were reduced by 95% (*p* < 0.0001) in the doxycycline group, but there was no significant change in the placebo group (*p* = 0.2588).

Microfilaremia was only slightly, though not significantly, reduced 4 mo after doxycycline treatment compared to pretreatment levels in the doxycycline group (*p* = 0.2741) whereas microfilaria levels had actually increased in the placebo group (*p* = 0.0017). Both doxycycline and placebo patients were given albendazole and ivermectin after the 4 mo re-examination to clear microfilaremia. At 12 mo post therapy, microfilaremia was virtually absent in the doxycycline group except in one of the 17 (6%) patients, who had a very low MF count. At 24-mo follow-up, 18% of doxycycline-treated patients that were re-examined had microfilaremia. In contrast, 50% of placebo patients became microfilaremic at 12 mo, and the number increased to 63% at 24 mo. The reduction of MF in the doxycycline group compared to placebo group was significant at the 12 mo (*p* = 0.0029) and 24 mo (*p* = 0.0211) follow-up time points. It must be mentioned that all patients continued to live in the endemic area; given a 5-y average life-span of adult worms and constant worm loads without treatment interference, new infections are expected to occur at a rate of about 20% per year.

### Measurement of Adult Worm Vitality in Microfilaremic Patients

Circulating filarial antigen (CFA) levels in plasma, and presence or absence of worm nests in ultrasound examinations, were used to assess macrofilaricidal activity of doxycycline. CFA did not differ significantly between the two groups before and at 12 mo after treatment (*p* = 0.5718 and *p* = 0.1748, respectively). However, the difference became significant at 24 mo post therapy (*p* = 0.0382), with median CFA levels in the doxycycline group being only 6% of pretreatment (Table 1). Before doxycycline treatment, all the 33 (17 doxycycline and 16 placebo) patients had antigenemia levels higher than 32,000 units (highest titer ranking according to the manufacturer, TropBio). However, 5/17 (29%) and 11/17 (65%) doxycycline-treated patients had antigen units lower than the 32,000 units at 12 and 24 mo, respectively, after treatment, whereas only 3/16 (19%) and 4/16 (25%) of placebo patients had antigens lower than 32,000 units at these time points. Ultrasonography to detect the filarial dance sign (FDS) as a second parameter of adult worm vitality was performed only in male patients since our earlier data [46] had shown that the FDS is detected less frequently and probably less reliably in women. FDS in the scrotal region before treatment showed that nine of 13 male patients examined in the doxycycline group had between 1–4 worm nests, whereas ten of 11 male patients in the placebo group had worm nests ranging from 1–5 worm nests. Considering only those male patients who had detectable FDS before treatment (nine doxycycline and ten placebo), 67% (6/9) and 89% (8/9) of male doxycycline-treated patients became FDS-negative at 12 and 24 mo after treatment, respectively. In contrast, all ten male placebo patients remained FDS-positive at 12 mo and 20% (2/10) were FDS-negative at 24 mo post therapy. There was no difference between the two groups before treatment (*p* = 0.3271); however, the difference became significant at 12 and 24 mo after doxycycline treatment (*p* < 0.001, Table 1).

### Secondary Outcome Variables in Microfilaremic Patients

#### Dilation of supratesticular lymphatic vessels.

**Figure 2:**
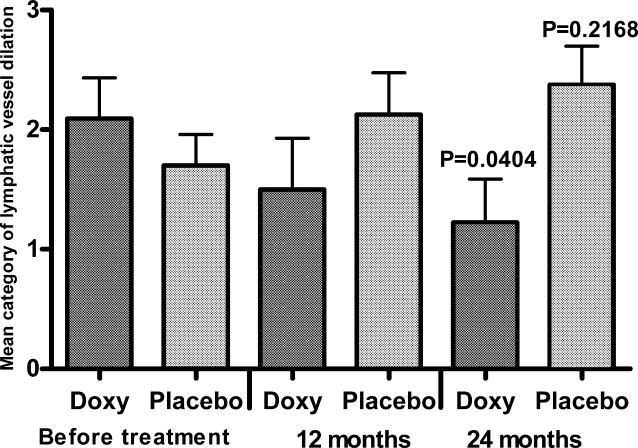
The Effect of Doxycycline Treatment on the Supratesticular Lymphatic Vessel Dilation at Various Time Points The supratesticular lymphatic vessel dilation (mean of category ± SD) was determined before treatment, and 12 and 24 mo thereafter, using USG. The mean supratesticular lymphatic vessel dilation of doxycycline-treated (Doxy) patients improved significantly compared to pretreatment (*n* = 9, *p* = 0.0404) at 24 mo, in contrast to the placebo group (*n* = 8) (paired *t*-test).

Before doxycycline treatment, ultrasonography (USG) showed that 17 male patients (nine doxycycline and eight placebo-treated) had supratesticular lymphatic vessel dilation. Figure 2 and Table 2 illustrate changes, after doxycycline treatment, in the dilation of lymph vessels that had not contained worms at study onset. Dilated lymphatic vessels containing no worms were reduced significantly at 24 mo after treatment (*p* = 0.0404) in the doxycycline group but not in the placebo group (*p* = 0.2168). Of the nine doxycycline-treated patients, seven (78%) patients showed a reduction of the lymphatic vessels in comparison to 1/8 (13%) patients in the placebo group (*p* = 0.0319, Table 2). In addition, lymphatic vessels that had contained adult worms at study onset were also reduced to normal in the doxycycline group at 24 mo.

**Table 2:**
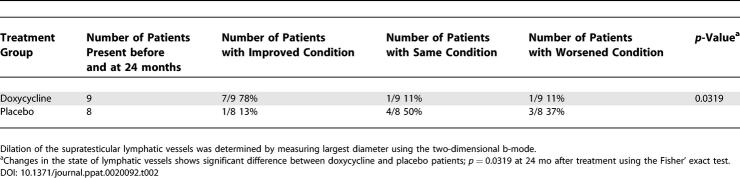
State of Lymphatic Vessel Dilation of Patients before and 24 Months after Doxycycline Treatment

#### Incidence of hydrocele during the 24-mo observation period.

In support of the hypothesis that doxycycline can ameliorate lymphatic vessel alteration, more patients in the placebo group developed a hydrocele during the observation period compared to the doxycycline group. Thus, one patient in the doxycycline-treated group developed a clinical hydrocele whereas in another patient, the hydrocele disappeared. In contrast, in the placebo-treated group, five patients developed hydrocele (three subclinical, as assessed by USG, and two clinical). This increase of hydrocele in the placebo group was significant when compared to the pretreatment status (hydrocele positive/negative at pretreatment: 1/10; at 24 mo: 6/5, *p* = 0.0221, chi-square test).

#### Lymphangiogenic factors.

**Figure 3:**
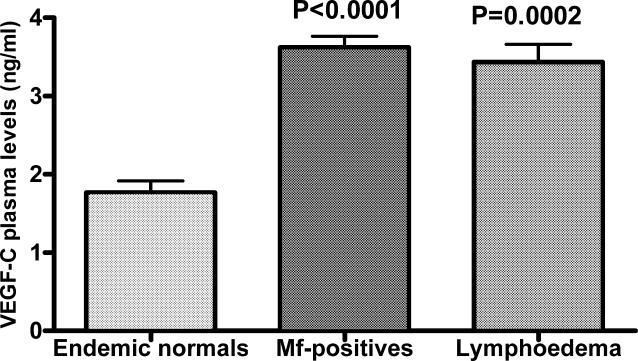
Pretreatment Plasma Levels of VEGF-C in Filarial-Infected Patients and Endemic Controls Plasma concentrations (mean ± SD) of VEGF-C were measured, using a commercial kit, from plasma of lymphedema patients (*n* = 26), microfilaremic patients (*n* = 76), and endemic controls (*n* = 23, who did not have filarial infection). Mean plasma levels of VEGF-C were significantly elevated in the microfilaremic (*p* < 0.0001) and lymphedema patients (*p* = 0.0002) compared to endemic controls (Student *t*-test with Bonferroni/Dunn correction). There was no difference between microfilaremic and lymphedema patients (*p* = 0.8033).

**Figure 4:**
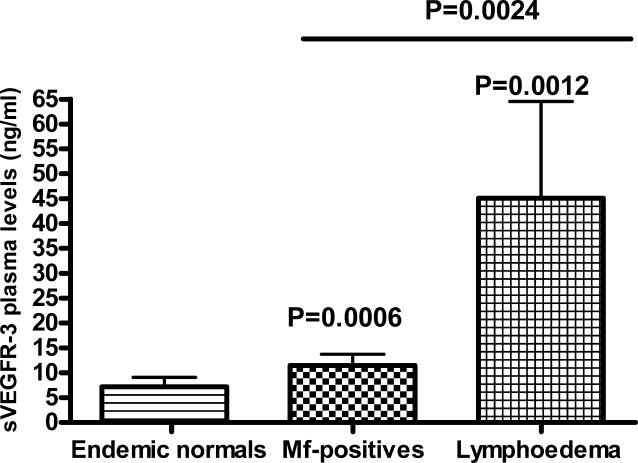
Pretreatment Plasma Levels of sVEGFR-3 in Filaria-Infected Patients and Endemic Controls Plasma concentrations (mean ± SD) of sVEGFR-3 were measured using a commercial kit from plasma of lymphedema patients (*n* = 26), microfilaremic patients (*n* = 76), and endemic controls (*n* = 23, who did not have filarial infection). Mean plasma levels of sVEGFR-3 were significantly elevated in the microfilaremic (*p* = 0.0006) and lymphedema patients (*p* = 0.0012) compared to endemic controls (Student *t*-test with Bonferroni/Dunn correction). sVEGFR-3 was also significantly elevated in lymphedema patients (*p* = 0.0024) compared to microfilaremic patients.

**Figure 5:**
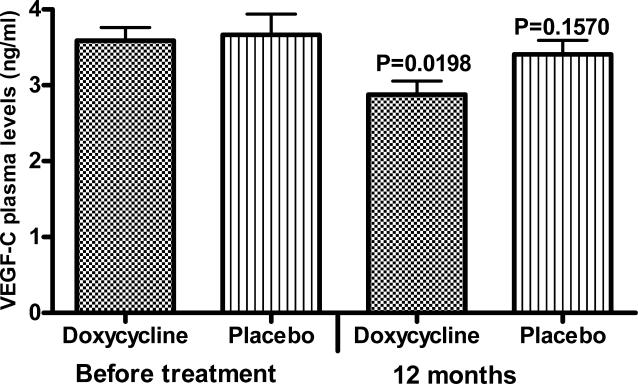
Plasma Levels of VEGF-C of Microfilaremic Patients before and 12 Mo after Doxycycline Treatment Plasma concentrations (mean ± SD) of VEGF-C were measured from plasma of microfilaremic patients before and 12 mo after doxycycline treatment (17 doxycycline treated, 16 placebo, see Table 1). The VEGF-C levels decreased significantly at 12 mo (preceding supratesticular lymphatic dilation, see Table 2) in the doxycycline-treated patients (*p* = 0.0198), but no difference in the placebo group occurred (paired *t*-test).

**Figure 6:**
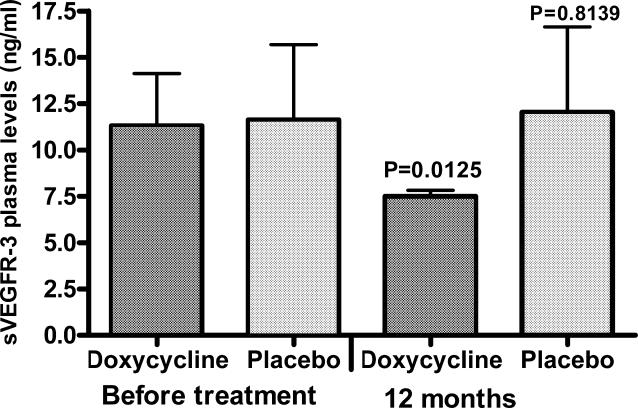
Plasma Levels of sVEGFR-3 of Microfilaremic Patients before and 12 Mo after Doxycycline Treatment Plasma concentrations (mean ± SD) of sVEGFR-3 were measured from plasma of microfilaremic patients before and 12 mo after doxycycline treatment (17 doxycycline treated, 16 placebo, see Table 1). The sVEGFR-3 levels decreased significantly at 12 mo (preceding supratesticular lymphatic dilation, see Table 2) in the doxycycline-treated patients (*p* = 0.0125) to a level close to that of endemic controls whereas there was no difference in the placebo group (paired *t*-test).

Mean plasma concentrations of VEGF-C and soluble VEGFR-3 (sVEGFR-3) were significantly elevated in microfilaremic patients (*p* < 0.0001, Figure 3, and *p* = 0.0006, Figure 4, respectively) compared to endemic controls, i.e., residents of the same endemic area with no evidence of infection despite exposure to infective larvae (see also Materials and Methods). Following doxycycline treatment, mean values of VEGF-C in the doxycycline group were significantly reduced (*p* = 0.0198, Figure 5), whereas there was no significant reduction in the placebo group. Mean plasma values of sVEGFR-3 in the doxycycline group were also significantly reduced 12 mo after doxycycline treatment to a level close to that of the endemic normals (*p* = 0.0125, Figure 6). In contrast, mean levels remained almost the same in the placebo patients. Samples from 24 mo post therapy were not sufficient in volume for VEGF-C and sVEGFR-3 analysis because most patients refused to donate the usual 10 ml of venous blood.

#### Associations between plasma levels of growth factors and parasitological parameters.

Using pretreatment samples, we found a positive correlation between VEGF-C and sVEGFR-3 (*r* = 0.430, *p* = 0.0014). No correlation was found between VEGF-C and lymphatic dilation, but a strong trend (*r* = 0.390, *p* = 0.0514) was noticed between sVEGFR-3 and lymphatic vessel dilation. There was also a positive association between levels of VEGF-C and antigenemia (*r* = 0.434, *p* < 0.0001) and between levels of sVEGFR-3 and antigenemia (*r* = 0.367, *p* = 0.0065), but no associations between microfilaremia and levels of VEGF-C (*p* = 0.8864) and sVEGFR-3 (*p* = 0.7720) were found.

### Primary Outcome Analysis in Lymphedema Patients

#### CFA and antifilarial antibody titers of lymphedema patients.

Twenty-six lymphedema patients were recruited (Figure 1B), and 19 met the inclusion criteria. Of the 19 patients, one patient was absent at 12 mo, leaving 18 patients who were present before and 12 mo after treatment. Of the 18 patients, eight received doxycycline and ten received placebo (Figure 1B). All lymphedema patients were CFA-positive except two (one doxycycline- and one placebo-treated) patients who had CFA levels below the cut-off of 128 units/ml; these two patients however had positive filarial antibody titers (all subclasses are measured in this test). One placebo patient was microfilaremic. All 18 patients had filarial antibody titers (1:40–1:640).

There were reductions in the antigenemia levels in both doxycycline and placebo patients with lymphedema at 12 mo, but no significant difference in the reduction was obtained in any of the groups (Table 3) at this time point. The antibody levels were significantly reduced in the doxycycline-treated group compared to pretreatment (*p* = 0.0431, Wilcoxon rank test), whereas there was no significant difference in the placebo-treated group (*p* = 0.0935, Wilcoxon rank test).

**Table 3:**
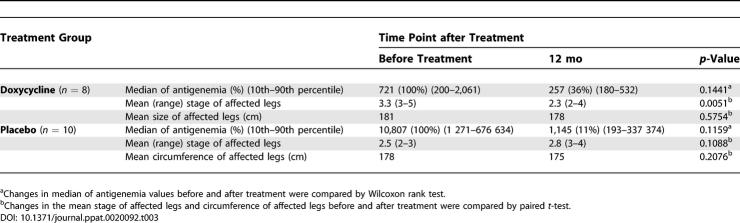
Primary Variables Measured before and after Treatment in Lymphedema Patients

#### Stages of lymphedema.

Table 3 shows the changes in the lymphedema stage—determined according to the staging scheme by Dreyer et al. [47]—of the affected legs and the circumference of the affected legs from baseline and 12 mo after treatment. The affected legs of all the doxycycline-treated patients reverted to a lower lymphedema stage at 12 mo post therapy, whereas all the placebo-treated patients stayed the same or changed to a higher stage at this time point. There was a significant reduction (*p* = 0.0051, Wilcoxon rank test) in the lymphedema stage of the doxycycline-treated patients at 12 mo post therapy compared to pretreatment, but not in the placebo group). The reduction in the stage manifested as better skin integrity (fewer “knobs”), reduction of deep and shallow skin folds, and fewer entry lesions of the affected legs (not shown). No change in the mean circumference of the affected legs in both doxycycline and placebo groups at 12 mo post therapy was observed.

#### Lymphangiogenic factors of lymphedema patients.

**Figure 7:**
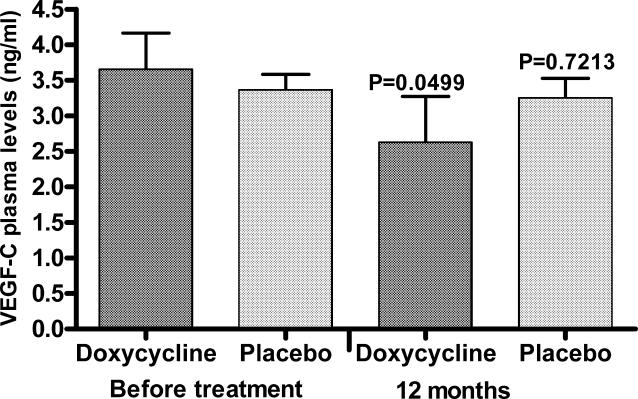
Plasma Levels of VEGF-C of Lymphedema Patients before and 12 Mo after Doxycycline Treatment Plasma concentrations (mean ± SD) of VEGF-C were measured from plasma of lymphedema patients before and 12 mo after doxycycline treatment. The VEGF-C levels decreased significantly at 12 mo in the doxycycline-treated patients (*p* = 0.0499), in contrast to the placebo group (paired *t*-test).

**Figure 8:**
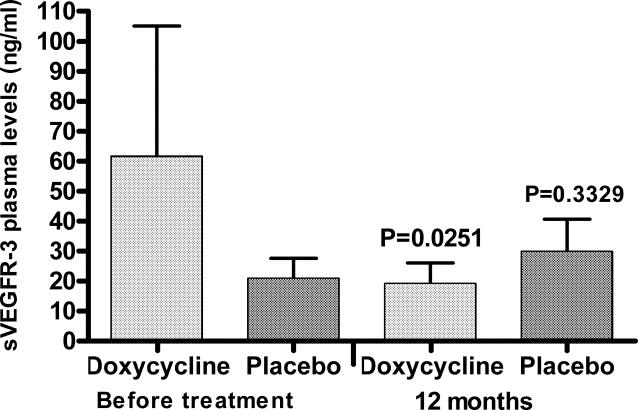
Plasma Levels of sVEGFR-3 of Lymphedema Patients before and 12 Mo after Doxycycline Treatment Plasma concentrations (mean ± SD) of sVEGFR-3 were measured from plasma of lymphedema patients before and 12 mo after doxycycline treatment. The sVEGFR-3 levels decreased significantly at 12 mo in the doxycycline-treated patients (*p* = 0.0251) whereas there was no difference in the placebo group (paired *t*-test).

Mean plasma concentrations of VEGF-C and sVEGFR-3 (Figures 3 and 4) were significantly elevated in lymphedema patients (*p* = 0.0002, and *p* = 0.0012, respectively) compared to endemic controls. Interestingly, the plasma levels of sVEGFR-3 were significantly higher in lymphedema patients than in microfilaremic patients without lymphedema (*p* = 0.0024, Figure 4). Twelve months following doxycycline treatment, mean values of VEGF-C and sVEGFR-3 in the doxycycline group were significantly reduced (*p* = 0.0499, Figure 7, and *p* = 0.0251, Figure 8, respectively), whereas there were no significant alterations in the placebo group. Since the pretreatment standard deviation of the sVEGFR-3 levels in the doxycycline group was rather large, due to one very high value, we also applied the Wilcoxon test in this case; there was still a significant difference (*p* = 0.0284) in sVEGFR-3 levels between pretreatment and 12 mo.

## Discussion

The past decade has seen major advances that have changed LF from a neglected disease into a disease now accepted as potentially eradicable. The main reason is the identification of ivermectin, DEC, and albendazole, as effective antifilarial agents.

While DEC has been shown to have some macrofilaricidal effect, it is still believed that a more potent macrofilaricide will add to efforts to achieve LF elimination, halting also the progression of the clinical manifestations induced largely by the adult worm [48]. This particularly is the case for Africa, where the use of DEC is discouraged in areas co-endemic for onchocerciasis, due to the irreversible ocular damage that DEC induces, different from ivermectin [49,50].

Two major advances have been observed in this study. Firstly, a regimen of 6 wk of doxycycline against *W. bancrofti,* shorter than what we published earlier [42], depleted *Wolbachia* endosymbionts and showed a strong macrofilaricidal activity, as evidenced by reduction of antigenemia by 94% and the absence of adult worms from the scrotal region in 89% of male participants by USG 24 mo after treatment. The second and fully novel advance is the reduction of lymphangiogenetic factors following doxycycline, and the reduction of lymphatic vessel dilation and improvement of lymphedema.

### *Wolbachia* Load

We recorded a 95% reduction of *Wolbachia* load in doxycycline-treated patients 4 mo after treatment, and this reduction was sustained throughout the 24-mo follow-up period. The fact that there was an apparent, but not significant, increase in *Wolbachia* loads in three MF-positive doxycycline patients at 24 mo is probably due to new infections, which is likely to occur in this area of ongoing transmission, theoretically at a yearly rate of 20% of the total worm load, given that the average worm life span is about 5 y and that there is a rather stable adult worm load in adult patients. New infections with concurrent rise in *Wolbachia* levels following doxycycline treatment have already been documented for doxycycline treatment of onchocerciasis, where old, doxycycline-treated and thus *Wolbachia* depleted, female worms were located in onchocercomas next to young, nulliparous worms that were full with *Wolbachia* [51]. Due to the unavailability of adult worms for histological and PCR analysis in LF, we could not formally confirm these findings for LF. Doxycycline treatment also resulted in almost complete elimination of microfilaremia which was sustained throughout the 24-mo period. This is consistent with our previous study [44]. The loss of microfilaremia in doxycycline patients is most likely due to the effect of *Wolbachia* depletion on embryogenesis and loss of microfilariae from host blood through natural attrition, as recorded in onchocerciasis [52,53] and lymphatic filariasis [42,44].

### Antigenemia

Even though antigenemia levels were significantly reduced by 94% in doxycycline-treated microfilaremic as compared to 48% in placebo patients, antigen units remained elevated at 24 mo in some doxycycline patients, though USG results showed the absence of worm nests. Before doxycycline treatment, all 33 (17 doxycycline and 16 placebo) patients had antigenemia levels above 32,000 units (the highest level that can be semiquantitatively determined according to the manufacturer, TropBio). However, at 24 mo, 11/17 (65%) of doxycycline-treated patients had antigen units below 32,000, compared to only 4/16 (25%) of placebo patients. Given that USG demonstrated absence of worm nests in most doxycycline-treated patients analyzed, our data suggest that antigenemia is cleared from the blood slowly, but progressively, after the death of the adult worms, as suggested by others [54]. The lower proportion of patients with high antigenemia in the doxycycline group underscores this and shows that it probably takes more than 24 mo for the antigens to be cleared from the blood after the death of the adult worms.

We cannot exclude that the partial reduction in antigenemia observed in the placebo patients could be due, to some extent, to a partial macrofilaricidal effect of ivermectin plus albendazole treatment. USG results did show 20% loss of worm nests at 24 mo; however, this reduction did not reach statistical significance. The lack of significance could be due to the rather small number of placebo patients (*n* = 16); however, even if a significant difference had been detected with higher numbers, the extent of the reduction would not have increased. Thus, our findings rather support other reports that ivermectin plus albendazole has no significant macrofilaricidal effect [55–57]. Another explanation that probably accounts for more of the reduction of antigenemia in the placebo group is the depletion of microfilaremia as a result of the antifilarial treatment offered to all the patients at the 4-mo time point. In this study, we found a positive correlation between microfilaremia and antigenemia (*r* = 0.754, *p* < 0.0001). This is consistent with another study [58] in which a positive correlation between microfilaremia and antigenemia was also reported, implying that as MF in the blood are depleted, the level of antigenemia also goes down, hence the reduction seen at 12 and 24 mo after treatment in the placebo group. A significant macrofilaricidal effect of ivermectin and albendazole in placebo patients is, however, not supported by the USG results, which demonstrated mostly stable worm nests in placebo patients (confirming earlier results by Dreyer et al. and others) [47,48], in contrast to most of the doxycycline-treated patients.

### VEGF Levels

Apart from the macrofilaricidal effect by doxycycline, the second observation in this study is that plasma levels of VEGF-C and its soluble receptor, sVEGFR-3, are significantly elevated in patients infected with filarial worms, and there is a correlation between sVEGFR-3 and lymphatic dilation. Targeting the filarial worms by doxycycline reduces the levels of VEGF-C/sVEGFR-3, with amelioration of dilated supratesticular lymphatic vessels and improvement in the conditions of lymphedema patients (Table 2, Figure 2). Given that the sVEGFR-3 are secreted into the plasma following overstimulation of the lymphangiogenesis system [59,60], these data indicate that the stimulation of lymphangiogenesis followed by lymphatic dilation may be reduced by doxycycline, and the VEGF-C/VEGFR-3 system may constitute a major mediator of pathological lymphatic dilation. This may be similar to what pertains in animal models in which inhibition of the binding of VEGF-C to membrane-bound VEGFR-3 by sVEGFR-3 led to complete destruction of the lymphatic network and a lymphedema-like phenotype [36]. Recent studies in solid tumor murine models have also correlated increased tumor-derived VEGF-C with lymphangiogenesis and lymph metastasis [61], supporting a role of VEGF-C in tumor progression by acting on lymphatic endothelium. It has also been shown that VEGFR-3 and its ligand VEGF-C are up-regulated in several diseases such as AIDS-linked Kaposi's sarcoma [62] and tumor lymphangiogenesis in breast cancer [63].

The mean plasma level of VEGF-C given by the manufacturer of Quantikine immunoassay enzyme-linked immunosorbent assay (ELISA) kits (R&D Systems, Wiesbaden, Germany) in a cohort of healthy European individuals is 225 (185–1,231) pg/ml. The levels of endemic normals in the present study was somewhat higher, with a mean of 1,851 pg/ml (range 634–2,805); it is not clear whether this reflects genetic differences between people of European descent and Africans, or a possible exposure of these persons to stimuli of the lymphangiogenetic system other than lymphatic filariasis. However, the levels of VEGF-C in microfilaremic and lymphedema patients were significantly increased over those of endemic normals before treatment. This finding, and more so the yet significantly elevated levels of sVEGFR-3 in lymphedema patients in comparison to microfilaremic patients (e.g., those with filarial infection but without overt lymphatic pathology), raises the question about the biological significance. Despite a vast literature on VEGF-C or sVEGFR3 expression in tissue (quantitative PCR and immunohistology), few data on plasma exist for these two factors, which are the major axis specific for lymphangiogenesis. What can be concluded from the available data are the following: (1) serum levels of VEGF-C are usually 10-fold higher than plasma levels, because of release from platelets during coagulation; this also means that plasma levels would be the more reliable marker since there is no (dominant) interference by platelet-derived VEGF-C [64]; and (2) measurement of plasma VEGF-C levels in cancer patients revealed 3-fold higher levels in comparison to controls [65]. In this regard, it is assumed that the almost 3-fold increase of plasma VEGF-C levels in our LF patients over endemic normals would be biologically significant based on the comparison with cancer patients. Importantly, in our study, VEGF-C levels decreased by 12 mo after doxycycline treatment, well before the supratesticular lymphatic dilation improved. The fact that the VEGF-C reduction preceded the improvement of pathology indicates a possible causal interaction between lymphangiogenic factors reduced by doxycycline treatment and lymphatic pathology, rather than only a coincidence or an epiphenomenon.

A report on VEGF-C levels in cervical cancer patients provides levels in serum, not plasma, and is therefore not directly comparable to our data (see the constraints above); nevertheless, in that report, after anticancer therapy the levels decreased to the level of controls, accompanied by concomitant improvement in the conditions of the patients [66], as also observed in our study in which levels decreased to those of endemic normals following doxycycline treatment.

Unfortunately no data exist as yet with regard to plasma sVEGFR3 levels and their biological significance for tumors. However, the more than 3-fold elevation in lymphedema patients is not considered to be biologically meaningless.

### Supratesticular Lymphatic Dilation

The degree of lymphatic dilation caused by filarial worms is considered an indirect measurement of the altered lymphatic function [67]. One study suggests that diffusible secretory products released either by the adult worm or by the human host in response to the parasite may induce lymphatic dilation [68]. Here, we have provided circumstantial evidence for both, in a series of events: the lymphatic dilation may be partly caused by overexpression of VEGF-C/sVEGFR-3 produced by the endothelial cells of the lymphatic vessels of the host in response to products of the adult worm that are reduced by doxycycline. Importantly, the reduction of dilated vessels was not mechanical as a result of the death of the adult worms, since the dilated vessels that were analyzed for this purpose did not contain worm nests.

The mechanism of action of doxycycline on the reduction of VEGF-C/sVEGFR-3 and its ameliorating effect on lymphatic vessel is not yet fully clarified. However, this may be associated with depletion of *Wolbachia* from adult filarial worms in the lymphatic vessels. There are now valid data from human studies that *Wolbachia* stimulate proinflammatory cytokines such as TNF, IL-1B, IL-6, and nitric oxide [45], which are known to up-regulate the expression of VEGF-C; importantly, these cytokines are down-regulated after doxycycline treatment [45]. This raises the possibility that proinflammatory cytokines such as TNF and IL-1B induced by *Wolbachia* could affect lymphatic vessels via VEGF-C and its receptor sVEGFR-3. It is therefore conceivable that any therapeutic intervention that causes reduction of VEGF-C–inducing proinflammatory cytokines such as TNF, IL-1B [45], etc. would be able to reduce levels of VEGF-C/sVEGFR-3, and hence lead to reduction of dilated supratesticular lymphatic vessels as we have shown here.

In this regard, it is remarkable that there was a significant increase in the prevalence of hydroceles in placebo as compared to doxycycline treated patients in our study.

### Lymphedema Stages

Doxycycline treatment significantly reduced the stage of disease in lymphedema patients, whereas the stage was not significantly altered in placebo patients, who, rather, showed a trend towards deterioration. The improvement in the stage in doxycycline patients manifested as better skin texture, fewer deep folds, and also fewer entry lesions of the affected legs (unpublished data). This suggests that doxycycline can be used to improve the clinical manifestations of lymphedema due to filariasis. A reduction of the number of adenolymphangitis (ADL) attacks that was noted in both, doxycycline and placebo patients (unpublished data) could be attributed to the foot-care hygiene training given to all the lymphedema patients, and with an attempt by patients to demonstrate best compliance. This is consistent with other reports [8,10,69,70], which also showed a significant reduction of attacks due to foot-care hygiene. However, a combination of foot-care hygiene and DEC gave no additional benefits regarding an improvement of the lymphedema stages [8,69,70]. This confirms that local limb care is an important intervention, whereas addition of doxycycline further reduces the severity of lymphedema. Neither doxycycline nor placebo patients showed a significant reduction of the circumference of the affected legs. This may be due to the fact that that circumference determination is not a reliable parameter to assess improvement of the disease, as suggested by Dreyer and co-workers [47], since it shows considerable variability due to transient effects such as keeping the leg elevated in the hours before measurement, or the female monthly hormonal cycle, etc. [47].

Clinical manifestations of lymphedema do not only hasten the progression of lymphedema to elephantiasis [71], but also reduce the workforce and economic resources of the affected individuals and communities [72,73] in many endemic areas. Therefore, better treatment options are mandatory.

The current treatment of lymphedema, which mainly relies on foot hygiene, is suboptimal and still empirical. This is because it is not clear at present to which extent ADL attacks are caused by either filarial worms, including *Wolbachia* endosymbionts, by exogenous bacteria, or by both [74,75].

Several chemotherapeutic agents have been tested, but so far, only 5,6 benzo-alpha-pyrone (coumarin) showed some encouraging results [76]. However, this drug is no longer recommended since it has been shown to be hepatotoxic [77]. Penicillin, which acts on some exogenous bacteria such as streptococci, but not on *Wolbachia* endosymbionts, has also been tested and reported to be beneficial in reducing only the incidence of ADL attacks [8], but there was no significant reduction in the lymphedema stages [8]. In contrast, in our study, treatment with doxycycline led to a significant amelioration of lymphedema stages. This could be due to the fact that doxycycline, in addition to targeting exogenous bacteria, also targeted *Wolbachia* endosymbionts and reduced lymphangiogenic factors along with a reversion of lymph vessel dilation. In order for doxycycline to exert its ameliorating activity by targeting *Wolbachia,* it is mandatory that the targets, i.e., adult worms or at least incoming L3/4 larvae, are present in the host's body. Indeed, all our patients were either CFA-positive (although most they did not exhibit high levels, as known for lymphedema patients) [78], or they showed clearly positive antifilarial antibody titers as a sign of recent exposure (two cases), in accordance with the assumption that it is a characteristic feature of lymphedema that the patients have a strong immune reaction that is targeted to incoming L3 and L4 larvae, effectively killing them, however at the expense of strong inflammation [21]. Studies are currently ongoing to compare the efficacy of doxycycline in CFA-positive in comparison to a larger group of CFA-negative lymphedema patients.

Lymphedema patients have a high level of suffering, and it can be well expected that they would use doxycycline for 6 wk without the need of a directly observed treatment (DOT). This is so all the more since the current mass treatment does not improve lymphedema per se. Hence, doxycycline treatment will have a good chance to be the first chemotherapeutic approach to address lymphatic pathology.

In conclusion, our data are in agreement with the hypothesis that progression of infection to lymphedema may be due to overexpression of lymphangiogenic factors such as VEGF-C and more so, sVEGF-R3, first due to the presence or the death of adult filarial worms in the lymphatic vessels and later (in the CFA-negative phase of the disease) either by the incoming larval stages of the parasite releasing *Wolbachia* upon being killed, or (additionally) by skin commensals such as streptococci [71,79,80] that exacerbate the condition by stimulating more proinflammatory cytokines and VEGF molecules, which are also reduced by doxycycline. This hypothesis is further supported by another study [81] in which the level of serum VEGF was shown to remain the same in patients with bancroftian filariasis after DEC treatment [81], which does not have beneficial effects on lymphedema patients apart from reducing parasite loads [8]. This is probably due to the fact that DEC treatment has no effect on bacteria (neither *Wolbachia* nor exogenous species), and therefore has no effect on proinflammatory cytokines that regulate this VEGF family, although it does partially reduce adult worm levels.

Although both microfilaremic and lymphedema patients had elevated VEGF-C and sVEGFR-3 levels, those of sVEGFR-3 were yet significantly higher in lymphedema patients compared to non-lymphedema patients that are microfilaremic (*p* = 0.0024, Figure 4). This could be an indication that plasma levels of VEGF-C/sVEGFR-3 may correlate with disease progression in LF leading to lymphatic dilation and lymphedema development and hence, might be developed as prognostic indicators of an increased risk of LF pathology before it actually becomes manifest. Importantly, reduction of lymphangiogenic factors preceded the improvement in pathology. On the one hand, these data argue against a possible hypothesis that elevated levels of these factors merely reflect infection. On the other, they do offer a potential to identify individuals who are prone to develop more severe disease. For this, a prospective study is needed that would screen VEGF-C and sVEGFR-3 levels in children and young adults, and monitor development of pathology such as lymphedema. In human studies of lymphedema families, heterozygous inactivating missense mutations have been detected in the tyrosine kinase–encoding region of Flt4 (VEGFR-3) [82], indicating that some lymphedema patients have dysfunctional lymphatics due to defective VEGFR-3 signaling. This might also be exploited for the development of an early marker for lymphedema.

Considering the possible effect of VEGF-C/sVEGFR-3 on lymphatic dilation and lymphedema development, a combination of classical antifilarial plus antiwolbachial therapy, which will reduce production of proinflammatory cytokines, may prove to be more effective in treating pathogenesis associated with LF than antifilarial therapy alone. This study represents a first observation that antiwolbachial therapy does not only have macrofilaricidal activity but may also halt progression of manifestations associated with lymphatic filariasis, and have a potential restorative effect on LF patients. As discussed earlier, compliance to take doxycycline for extended periods is not expected to be an issue with individuals suffering from lymphatic pathology.

## Materials and Methods

This placebo-controlled, double-blind study was carried out in the Nzema East District in the Western region of Ghana. The study was approved by the Ethical Committee on Human Research and Ethics of the School of Medical Sciences of Kwame Nkrumah University of Science and Technology (KNUST), Kumasi, Ghana, as well as by the ethics committee of the University of Liverpool which acted as a control body since this work formed part of a European study network funded by the European Commission. The study conformed to the principles of the Helsinki Declaration of 1975 (as revised in 1983 and 2000). The trial registration number is ISRCTN 14757.

### Study population.

Individuals enrolled from the neighboring villages of Adjan, Domunli, and Akonu in the Western Region of Ghana took part in the study. No other human filarial species were endemic in these villages. A total of 76 (55 males and 21 females) microfilaremic (Figure 1A) and 19 (13 females and six males) lymphedema (Figure 1B) patients were included in this study. The study site was selected based on an established occurrence of lymphatic filariasis within the surrounding region and clinical observations (rapid assessment) consistent with symptomatic disease in a proportion of villagers [44]. Written informed consent was obtained from all participants. Individuals eligible for participation were adults of both sexes aged 18–50 y, with a minimum body weight of more than 40 kg, in good health, and without any clinical condition requiring chronic medication. Hepatic and renal function and pregnancy were assessed by dipstick chemistry. Exclusion criteria encompassed a microfilarial load <50 MF/ml (microfilaremic patients), abnormal hepatic and renal enzymes (aspartate aminotransferase [AST; 0–40 IU/l], alanine aminotransferase [ALT; 0–45 IU/l] and creatinine [3–126 μmol/l]), pregnancy, lactation, intolerance to doxycycline, alcohol or drug abuse, or antifilarial therapy in the last 10 mo.

### Randomization of patients and treatment regimens.

Randomization of patients was carried out using computer-generated random number software (StatView). Blinding was assured by the exclusion of persons involved in randomization or tablet packaging in any clinical or laboratory assessment.

Participants received 2 × 100 mg capsules of doxycycline (Vibramycin; Pfizer, New York, New York, United States) or matching placebo supplied by the manufacturer daily for a total of 6 wk. Treatment was done and monitored by a trial clinician in the form of daily observed treatment (DOT). Four months after the start of treatment, all participants received a standard oral dose of 400 mg albendazole (GlaxoSmithKline, Uxbridge, United Kingdom) and 150–200 μg/kg ivermectin (Mectizan; Merck, Sharp & Dohme, Clermont-Ferrand, France).

### USG examinations.

**Figure 9:**
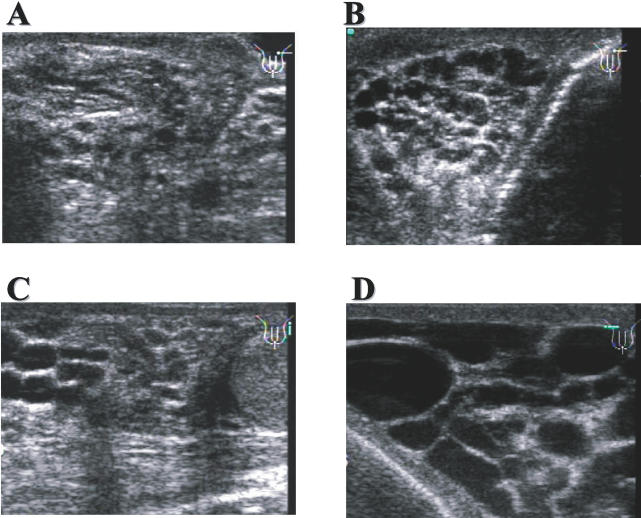
Grading of the Supratesticular Lymphatic Vessel Dilation of Filarial-Infected Patients Displayed by USG Dilation of the supratesticular lymphatic vessels was determined by measuring the largest diameter detectable in the two-dimensional b-mode of a portable ultrasound machine. A grading system was developed to determine the degree of lymphatic dilation as follows: (A) category 1: patients with minimal lymphatic dilation of up to 0.2 cm; (B) category 2: patients with mild dilation from 0.21–0.50 cm; (C) category 3: patients with moderate dilation from 0.51–1.0 cm; and (D) category 4: patients with severe dilation of above 1.0 cm.

Male participants were examined using a portable ultrasound machine (SonoSite 180 Plus; SonoSite, Bothell, Washington, United States) equipped with a 7.5 MHZ linear transducer as described previously [83]. Briefly, patients were screened for worm nests in the scrotal area. Each detected worm nest was documented using print outs in b-, m-, and pulse-wave Doppler modes. Additionally, worms in lymphatic vessels were recorded on DVtapes using a SonyPC 120E Handycam (Sony, Tokyo, Japan) connected to the ultrasound machine in order to get an animated documentation of the moving worms. Dilation of the supratesticular lymphatic vessels containing no worm nests was determined by measuring the largest diameter using the two-dimensional b-mode. Since there is no grading system for lymphatic dilation, we developed a grading system to determine the degree of lymphatic dilation as follows: category 0 = no dilation (normally up to 0.10 cm [84], category 1 = the maximally dilated vessel has a dilation up to 0.20 cm, category 2 = dilation from 0.21–0.50 cm, category 3 = dilation from 0.51–1.0 cm, and category 4 = dilation above 1.0 cm (Figure 9A–9D). The procedure was repeated 12 and 24 mo after treatment.

### Determination of microfilarial load.

For a quick screening in the night, the microfilarial load was determined by microscopic examination of finger-prick blood samples as published [44]. Subsequently, eligible patients donated 10 ml of venous blood for accurate quantification using Whatman Nucleopore filter method as described previously [44]. The same volume of blood was taken from each patient 4 and 12 mo after the commencement of doxycycline treatment, but a lesser volume of 7 ml was taken at 24 mo due to complaints from the patients. At each time point, plasma was taken from the remaining sample, aliquoted, and frozen at −80 °C for later analysis of antigenemia and lymphangiogenic factors.

### Determination of *Wolbachia* levels in MF by PCR.

*Wolbachia* content was quantified by real-time PCR of the *W. bancrofti Wolbachia*–*ftsZ* gene, derived from 500–1,000 microfilariae using a Rotor-Gene 3000 (Corbett Research, Brussels, Belgium) at pretreatment and 4, 12, and 24 mo after treatment. Details of the technique are given in reference [44]. Briefly, DNA was extracted with DNeasy kit (Qiagen, Hilden, Germany) following manufacturer's protocol with Proteinase K digestion. For the quantification, primers and a Taqman hybridization probe with the fluorescent dye 6-FAM (6-carboxyfluorescein) (Qiagen) were used to amplify a 286-base pair fragment of the *W. bancrofti Wolbachia*–*ftsZ*. The products were quantified by comparing with a standard curve of the plasmid containing the *W. bancrofti Wolbachia*–*ftsZ* fragment.

### Determination of circulating filarial antigenemia.

For determination of circulating filarial antigenemia (filarial adult worm antigens), W. bancrofti antigen was measured with the TropBio ELISA test kit (TropBio, Townsville, Australia). The manufacturer's protocol was followed except that the samples were diluted (1:20 ratio) with the diluent [42] before pipetting into the TropBio ELISA test plates. Samples were tested in duplicate before treatment and at 12- and 24-mo follow-ups. The optical density at 414 nm was recorded from plasma samples. Antigen units were calculated with a standard curve from standards provided by the manufacturer, and the final units multiplied by the dilution factor of 20.

### Limb measurement of lymphedema patients.

The circumference of both affected and normal legs were measured with measuring tape at five different points. Measurements were taken at 10 cm from the large toe, and 12 cm, 20 cm, 30 cm and 50 cm from the sole of the foot as described in reference [85], and the average of the five measurements taken as the mean circumference of the leg. Measurements were taken before and 12 mo post therapy.

### Grading or staging of lymphedema legs.

**Figure 10:**
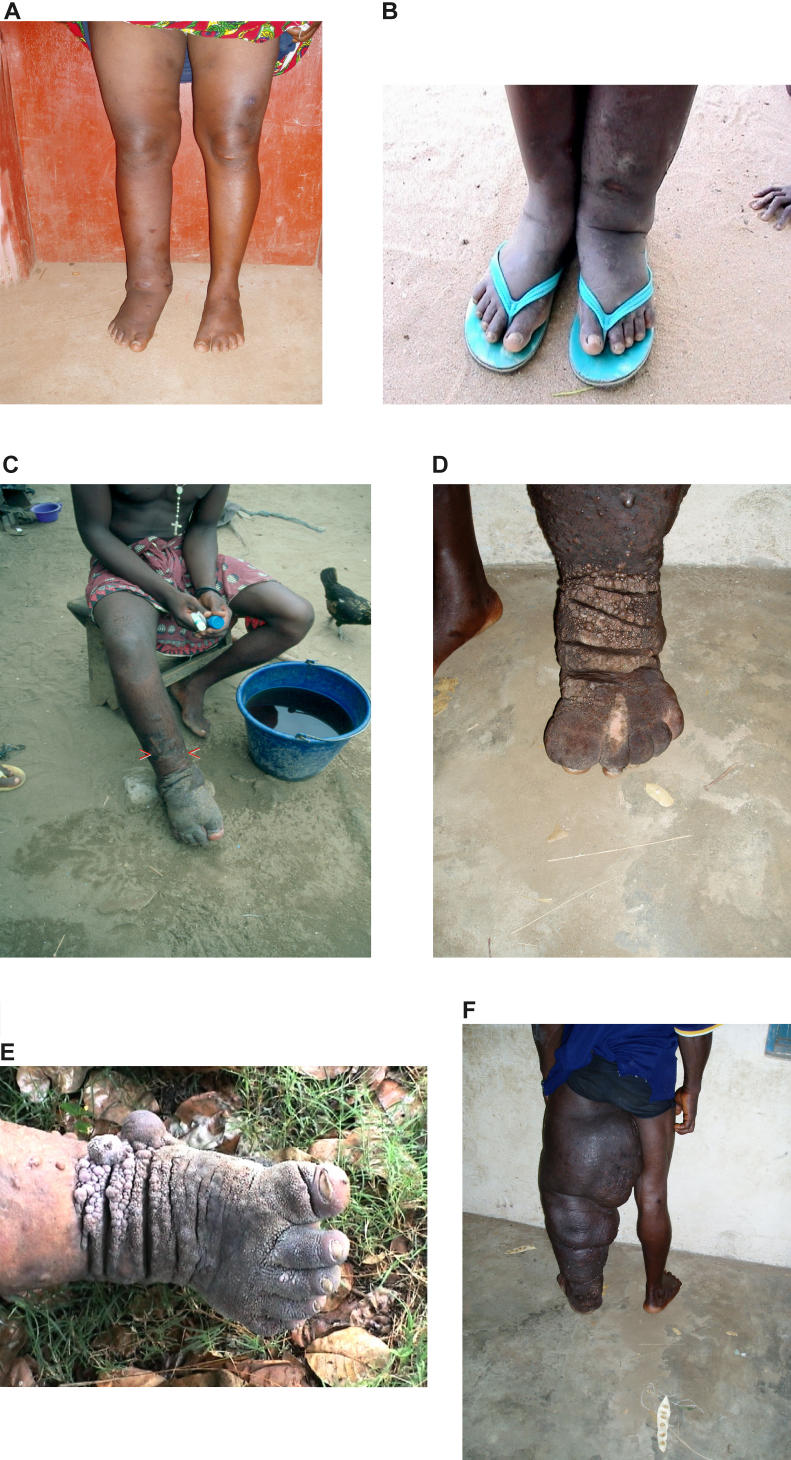
Lymphedema Stages The lymphedema stages according to the classification by Dreyer et al. [47]; patients are from this study. (A) Stage 2, swelling that is not reversible overnight. (B) Stage 3, shallow skin folds at the ankle. (C) Stage 4, alteration of skin texture and formation of knobs (arrowheads). (D) Stage 5, presentation of deep skin folds in addition to the alterations of stage 4; (E) Satge 6, presentation of mossy lesion in addition to the alterations of stage 5. (F) Stage 7, inability of patient to perform daily work.

Grading or staging of lymphedema was performed along the guidelines of “Basic Lymphedema Management” [47] (see Figure 10.)

Stage 1 = swelling that is reversible overnight, Stage 2 = swelling that is not reversible overnight, Stage 3 = shallow skin folds (ankle), Stage 4 = presence of knobs (lumps or protrusions), Stage 5 = deep skin folds plus knobs, Stage 6 = deep skin folds plus knobs and mossy lesions, and Stage 7 = parameters mentioned above plus patient is unable to perform routine daily activities.

### Foot care and ADL.

Before the treatment began, all lymphedema patients were taught about hygienic care and physical exercises for the affected legs, and importantly, daily cleansing of affected legs with soap and water, and keeping the affected leg dry between washes. All the lymphedema patients were given soap, towels, and plastic bowls for washing the legs. They were visited every 6 mo in their villages. At each visit, the patients were asked through a questionnaire the number of attacks experienced.

### Serology test.

Filarial antibodies were measured from all the lymphedema patients before and 12 mo after treatment using the indirect immunofluorescence antibody (IIFA) test. For antigen preparation, adult B. malayi males were recovered 6 mo post infection from Mastomys coucha. Parasites were washed and stored at 4 °C for 4 h. They were then placed in canine musculature, frozen, and 7-μm sections were prepared and transferred onto glass slides. Quality was controlled by using a defined internal control serum (titer 1:320). For the IIFA test, 100 μl of plasma was inactivated at 56 °C for 30 min, and diluted 1:10 in PBS (pH 7.2). Seven-fold serial dilutions to 1:1,280 were made from the initial 1:10 screening dilution. A 10-μl volume of each of the diluted samples was added to separate wells on slides containing the B. malayi antigens, and incubated at 37 °C in a humid chamber for 45 min. After the incubation, the IIFA slides were washed four times for 20 min (3× in 0.1M PBS and 1× in deionized water). A fluorescein-labeled anti-total human immunoglobulin (Fluoline-H; bioMerieux, Marcy l'Etoile, France) diluted 1:90 in PBS was applied to each well and incubated for 45 min at 37 °C in a humid chamber. The slides were washed four times for 20 min (3× in 0.1M PBS and 1× in deionized water). Excess water was removed and the slides dried, covered with glycerol (Euroimmun, Luebeck, Germany), and cover slides. Wells were observed with a immunofluorescence microscope (Ernst Leitz, Wetzlar, Germany). Fluorescence of either the complete worm section including mesenchym and cuticle or only mesenchym was defined as positive. Fluorescence of the mesenchym without cuticle was regarded as non-specific. A titer of nore than 1:80 (including cuticle fluorescence) was regarded as indicative for filarial infection. A titer equal to or less than 1:20 was regarded as negative. Positive and negative controls were included in the test. This test is validated in regular intervals within a German network of laboratories for quality control.

### Determination of plasma levels of VEGF-C and sVEGFR-3.

In all patients, the plasma concentrations of VEGF-C and sVEGFR-3 were measured using Quantikine immunoassay ELISA kits according to the manufacturer's instructions (R&D Systems). After stopping the reaction, plates were read at 450 nm and 540 nm with a microplate reader (SPECTRAmax 340PC; Sunnyvale, California, United States). Twenty-three endemic normals, i.e., residents of the same endemic area with no evidence of infection confirmed by the lack of mircofilaraemia and lack of circulating Og4C3 filarial antigen, despite exposure to infective larvae borne by mosquitoes, were included as controls.

### Data analysis.

*Wolbachia* loads in worm tissue and microfilaremia were summarized as geometric means (GM). Differences in GM at baseline and subsequent follow-ups were analyzed using the Wilcoxon signed rank and Mann-Whitney *U* tests. Changes in the degree of antigenemia were calculated as percentages from baseline and analyzed between treatment groups at subsequent follow-up time points by the Mann-Whitney *U* test. Proportions of MF-positive and MF-negative individuals as well as individuals showing the fFDS before and after treatment were compared using Fisher' exact tests. Plasma levels of the VEGF molecules were expressed as mean ± standard deviations (SD), and differences in the levels of the VEGF molecules between endemic normals, microfilaremic (MF-positive), and lymphedema patients were analyzed using analysis of variance (ANOVA) with the Bonferroni/Dunn post hoc test. Differences in the levels of the VEGFs within the treatment groups before and 12 mo after treatment as well as changes in lymphatic vessel dilation, stages of lymphedema, and the size of affected limbs of lymphedema before and after treatment were assessed using paired *t*-tests. Positive relationships between the disease states and the VEGF molecules were assessed by the Spearman' rank correlation test and simple regression analysis (coefficient of determination indicated as *r*). A two-tailed *p*-value lower than 0.05 was considered significant. Independence of data was tested using multivariate analysis. All analyses were done using StatView version X for Mac OS 9.5.

## Supporting Information

### Accession Numbers

The GenBank (http://www.ncbi.nlm.nih.gov/Genbank) accession numbers for the gene and gene products discussed in this paper are: soluble VEGFR-3 (NM_002020); VEGF-C (CAA63907); and *Wuchereria bancrofti Wolbachia*–*ftsZ* gene (AF081198).

## Abbreviations

ADL: adenolymphangitis
CFA: circulating filarial antigen
DEC: diethylcarbamazine
ELISA: enzyme-linked immunosorbent assay
FDS: filarial dance sign
GM: geometric means
IL: interleukin
LF: lymphatic filariasis
MF: microfilaria
SD: standard deviations
sVEGFR-3: soluble vascular endothelial growth factor receptor-3
TNF: tumor necrosis factor
USG: ultrasonography
VEGF: vascular endothelial growth factor
VEGFR-3: vascular endothelial growth factor receptor-3
